# Genomic, morphological and migratory patterns in recovering Atlantic salmon populations

**DOI:** 10.1007/s00027-026-01288-1

**Published:** 2026-03-30

**Authors:** Paolo M Moccetti, Domino A. Joyce, Jonathan D. Bolland, Jamie R. Dodd, Andy D. Nunn

**Affiliations:** 1 School of Environmental and Life Sciences, University of Hull, Kingston upon Hull, UK; 2 Energy and Environment Institute, University of Hull, Kingston upon Hull, UK; 3 Department of Environment Constructions and Design, Institute of Earth Sciences, University of Applied Sciences and Arts of Southern Switzerland, Via Flora Ruchat-Roncati 15, 6850 Mendrisio, Switzerland; 4 Evolutionary and Ecological Genomics Group, School of Environmental and Life Sciences, University of Hull, Kingston Upon Hull, UK; 5 Hull International Fisheries Institute, School of Environmental and Life Sciences, University of Hull, Kingston Upon Hull, UK

**Keywords:** Population recovery, Recolonisation, Population structure, Local adaptation, Acoustic telemetry, Morphometrics

## Abstract

Population recovery is a key goal for conservation, but our understanding of how genetic and phenotypic variation, including local adaptation, emerges in recently recolonised or recovering systems is limited. This complicates the conservation of fitness-related traits that support resilience and long-term population viability. We employed single nucleotide polymorphism markers, geometric morphometrics and acoustic telemetry to investigate the recovery of Atlantic salmon (*Salmo salar*) in a large river system, which began more than 40 years ago following presumed extirpation in the mid-1900s. We observed genetic differentiation between Atlantic salmon in different tributaries that may reflect natural recolonisation through straying and/or the recovery of remnant populations previously believed to be extinct. Fine-scale philopatry was found to maintain the genetic divergence between tributaries, as confirmed by genetic assignment and telemetry tracking of returning adults, with some individuals displaying ‘search’ behaviour across tributaries. Despite this population structuring, neither consistent genomic signatures of selection nor morphological divergence were detected, indicating no evidence for early stage local adaptation or for differing selective pressures between tributaries. The patterns instead align with stochastic demographic processes (i.e. founder effects and genetic drift), which was further supported by the low contemporary effective population sizes detected across tributaries. These findings highlight the importance of continued genomic and ecological monitoring during population recovery, as improving environmental conditions may promote the re-establishment of remnant populations and/or facilitate recolonisation. This may, in turn, lead to the formation of reproductively isolated units and, over time, the emergence of locally adapted traits of conservation value for Atlantic salmon and other threatened migratory species.

**Supplementary Information:**

The online version contains supplementary material available at 10.1007/s00027-026-01288-1.

## Introduction

Migratory species are declining globally due to environmental pressures such as habitat degradation and fragmentation, overexploitation, pollution, invasive species and climate change (United Nations Environment Programme World Conservation Monitoring Centre 2024). Despite the recognised importance of animal migration, including for ecosystems and human communities, our current understanding of such phenomena remains incomplete, hindering the development of effective conservation measures (Bowlin et al. 2010; Fudickar et al. 2021).

A central challenge in the study and conservation of migratory species is to understand how populations respond and adapt to the rapidly changing environments that they experience throughout their life cycles. Such responses can be expressed through a combination of genetic, phenotypic and behavioural changes, all of which can influence survival, reproductive success and long-term population persistence (Bernatchez 2016). In this context, management must aim not only to protect species, but also to preserve any locally adapted characteristics that increase the biological resilience and viability of populations (Flanagan et al. 2018; Funk et al. 2012). Consequently, there is an urgent need to harness newly available technological and analytical approaches, spanning genomics, phenotypic analyses and movement ecology, to increase our understanding of these processes. This will facilitate the conservation of migratory species as we mitigate growing threats, and restore and rewild habitats (Lennox et al. 2016).

Freshwater fish are among the most threatened of migratory taxa, surpassing other fish groups and terrestrial animals, with an estimated global decline of 81% between 1970 and 2020 (Deinet et al. 2024). Diadromous species, requiring free movement between marine and fresh waters to complete their life cycle, are especially at risk because they are exposed to multiple stressors in various environments, such as rivers, lakes, estuaries, coastal areas and the open ocean (Limburg and Waldman 2009; Costa et al. 2021). One notable diadromous species in decline is the Atlantic salmon (*Salmo salar* Linnaeus, 1758), a socioeconomically important fish that has experienced a dramatic reduction in numbers over the past four decades [International Council for the Exploration of the Sea (ICES) 2024] and is now classified as Near Threatened by the International Union for Conservation of Nature (Darwall 2023). In the most severe cases, this has culminated in the disappearance of the species from entire river systems (ICES 2024). Atlantic salmon are normally anadromous, i.e. after a few years in fresh water, they undertake an extensive feeding migration to the Atlantic Ocean before returning to fresh water after 1 or more years to spawn (Klemetsen et al. 2003). The round trip migration can cover thousands of kilometres (Strøm et al. 2018; Rikardsen et al. 2021), making Atlantic salmon particularly susceptible to multiple natural and anthropogenic stressors, including pollution, habitat fragmentation, escaped farmed fish, sea lice infections from aquaculture, harvesting, invasive species, and climate change (Forseth et al. 2017; Dadswell et al. 2022).

Importantly, Atlantic salmon display philopatric behaviour, meaning they normally home to their natal watercourse and nursery grounds to spawn (Thorstad et al. 2010; Jonsson and Jonsson 2011). Among the salmonids, although straying occurs, and can vary considerably across and within species and regions (Salmenkova 2017), Atlantic salmon usually show high fidelity to particular river systems, or even tributaries or specific areas within catchments. This limits gene flow between Atlantic salmon populations, causing many to be genetically and phenotypically unique (Garcia De Leaniz et al. 2007; Aykanat et al. 2015). This genetic and phenotypic differentiation often results in local adaptation, with the evolution of environment-specific traits positively linked to survival and reproductive success (Fraser et al. 2011). These include important life history, behavioural and physiological traits, such as age and size at maturity, migration timing, iteroparity and alternative reproductive strategies, which have repeatedly been found to be linked to genomic regions under divergent selection between populations, often in association with environmental gradients, spanning from within-river to among-continent scales (summarised by Pritchard et al. 2018; Mobley et al. 2021; Beck et al. 2025). A crucial aspect of Atlantic salmon conservation, therefore, is to detect and preserve these local adaptations by identifying intraspecific conservation units (Fraser and Bernatchez 2001).

Body morphology is a critical component of local adaptation in salmonid fishes, including Atlantic salmon, which exhibit substantial variation in traits such as head and mouth shape, fin size and body depth across river systems and habitat types (Riddell and Leggett 1981; Solem and Berg 2011; Drinan et al. 2012). This trait variation plays a major role in ecological interactions, influencing habitat use, feeding behaviour, predator avoidance, reproductive success and swimming performance (Skúlason and Smith 1995; Ardon et al. 2023). Because of its functional importance, body morphology is considered a key target of natural selection in ecologically driven diversification (Jonsson and Jonsson 2001; Skúlason et al. 2019) and can change rapidly in response to strong environmental pressures, whether through genetically based evolution or phenotypic plasticity (Hendry et al. 2000; Pakkasmaa and Piironen 2001; Haugen et al. 2008). Distinguishing whether morphological variation reflects genetically fixed adaptation or plastic responses is challenging, and often requires experimental or complex genomic approaches (Adams et al. 2003; DeLorenzo et al. 2023). However, whether such variation is genetically based or plastic, morphology provides an informative proxy for divergent selective pressures acting across environments, potentially leading to local adaptation (Taylor et al. 2011; Riesch et al. 2020).

While nearly all studies of adaptive divergence in Atlantic salmon have focused on large, historically stable populations, much less is known about how genetic and phenotypic variation, including local adaptation, emerges in recently recolonised or recovering systems. This topic is of increasing importance as the majority of Atlantic salmon populations continue to decline (Dadswell et al. 2022; Darwall 2023; Nunn et al. 2023), potentially resulting in severe size reductions and even local extirpations, followed by eventual recolonisation or recovery if conditions improve (Milner et al. 2008; Perrier et al. 2009). Importantly, adaptive shifts in salmonid traits have been shown to occur over only a few decades when selective pressures change (Haugen et al. 2008; Czorlich et al. 2022), suggesting that local differentiation, and potentially even early stage local adaptation, may already be occurring in some recovering populations (Sparks et al. 2023). Such cases are inherently complex, however, as evolutionary dynamics may be shaped by founder effects, genetic drift, and mixed ancestry from straying individuals or historical stocking events, and in some cases the persistence of low-abundance remnant populations (Grandjean et al. 2009; Griffiths et al. 2011; Ikediashi et al. 2012; Koed et al. 2020; Lennox et al. 2021). Understanding the relative influence of these processes is therefore essential for effective conservation and management, because failing to detect emerging population structure or early stage adaptation could result in inappropriate actions that compromise the persistence of locally adapted variation (Østergren et al. 2021).

The extent of genetic and morphological differentiation, potential genomic signatures of adaptive divergence, and homing behaviour in recovering Atlantic salmon populations in the Yorkshire Ouse (hereafter ‘Ouse’) catchment in northern England were examined in the present study. The Ouse is a large river system where the species was abundant and supported commercial fisheries until the early 1900s, but was considered to have been extirpated by the mid-twentieth century due to a combination of pollution, habitat degradation, overfishing, and unfavourable climatic conditions (Howes 1976; Axford 1991; Whitton and Lucas 1997). Although in Britain the Atlantic salmon is classified as Endangered with extinction (Nunn et al. 2023), the Ouse has been an exception to this declining trend and represents one of the few rivers with a recovering population(Cefas et al. 2021). This recovery appears to have begun in the 1980s, likely as a result of improvements in water quality and habitat restoration initiatives (Mawle and Milner 2003). Additionally, Atlantic salmon from Scotland were stocked into an Ouse tributary between 1965 and 1975 (Axford 1991), and locally captured brood stock were used to support a stocking programme in the 2010s. The Ouse's demographic history, coupled with the presence of large tributaries with suitable habitat potentially promoting local spatial differentiation (Ensing et al. 2011), makes it an excellent study system to explore genetic and phenotypic diversity, potentially underlying local adaptations, in recovering Atlantic salmon stocks, a few decades after the beginning of the species’ comeback.

Specifically, our objectives were to: (1) investigate the genetic structure and diversity of Atlantic salmon in tributaries of the Ouse to infer recent demographic and evolutionary processes; (2) determine whether individuals differ in body shape between tributaries, consistent with divergent selection and local adaptation; (3) determine whether the tributary populations exhibit genomic signatures of early stage adaptive divergence; and (4) assess whether adults, tracked during their upstream spawning migration, were strays of exogenous origin or successfully completed homing behaviour to their natal river, thereby potentially influencing patterns of population structure and local adaptation. We used genome-wide single nucleotide polymorphism (SNP) data to quantify population structure, genetic diversity and identify putatively adaptive loci, geometric morphometrics to assess phenotypic differentiation, and acoustic telemetry coupled with genetic analysis to assess individual homing behaviour and accuracy of immigrant fish. The results of such a comprehensive analysis provide an integrated assessment of population structure and diversity, adaptive divergence and migratory behaviour in a recovering system, with direct relevance for the conservation and management of Atlantic salmon and other declining diadromous species.

## Materials and methods

### Study area and stocking history

**Fig. 1:**
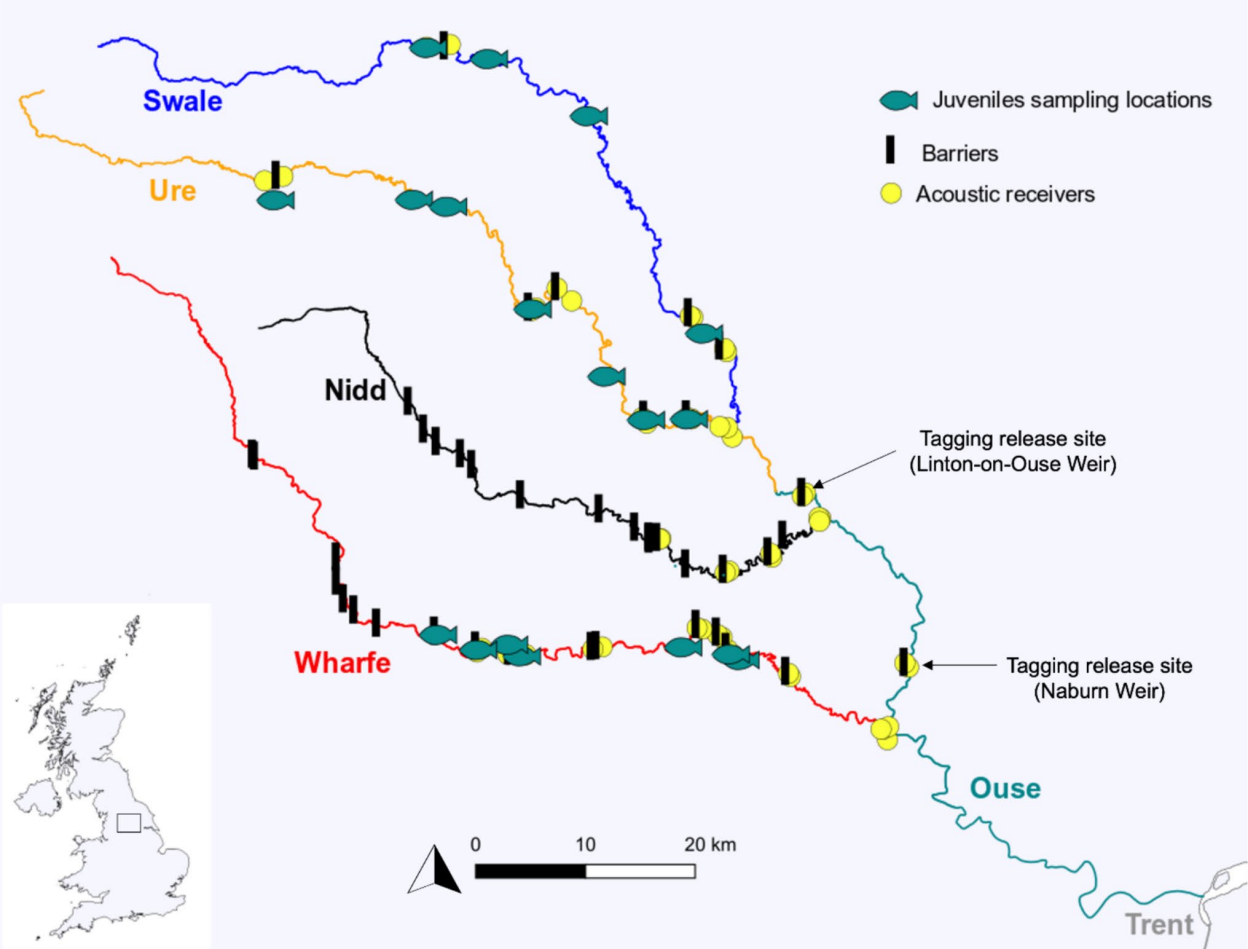
Map of the Yorkshire Ouse catchment showing the locations of juvenile Atlantic salmon sampling, potential migration barriers, adult tagging and acoustic receivers. Adults were sampled near the tagging sites. The map was created using QGIS version 3.4.12 (http://qgis.org)

This study was conducted in the Yorkshire Ouse catchment in northern England (Fig. 1). The Ouse is approximately 200 km long and drains a catchment of ~ 10,000 km^2^ before joining the River Trent to form the Humber Estuary. The Ouse catchment is predominantly rural and the quality of its water is generally good, but deteriorates in the downstream reaches due to the impacts of urban drainage, combined sewer overflows, sewage treatment works and inputs from the rivers Don, Aire and Foss (Jarvie et al. 1997; Lucas et al. 1998). The main stem of the Ouse lacks suitable spawning and nursery habitat for Atlantic salmon, resulting in their concentration within three of its major tributaries, namely the rivers Swale, Ure and Wharfe (Fig. 1). Of these, the Ure hosts the largest Atlantic salmon population (Mawle and Milner 2003) and is the only tributary that supports a recreational salmon fishery. Another tributary of the Ouse, the River Nidd, provides only small areas of sub-optimal habitat for the species and, as shown by historical surveys, rarely hosts Atlantic salmon.

Following the collapse of the populations, two stocking programmes were undertaken in an attempt to restore Atlantic salmon in the catchment. Notably, both initiatives focused exclusively on the River Ure, with the rivers Nidd, Swale and Wharfe not included. The first programme released approximately 1.8 million  *fry* (young fish that have recently begun feeding) originating from ova purchased from the River Tweed Commissioners and the Kyle of Sutherland Fishery Board in Scotland between 1965 to 1975 (Axford 1991). For the second stocking programme, conducted when Atlantic salmon numbers in the system were already increasing, brood stock captured in the River Ure were used, and approximately 200,000 hatchery-reared *parr* (i.e. older fish at the developmental stage before maturation) were released between 2014 and 2021.

### Juvenile fish

Electrofishing surveys were performed in 2019, 2020 and 2021 using a battery-powered backpack unit (Fig. 1). Where possible, a minimum of one site was selected in each river reach, i.e. between potential migration barriers on the rivers Swale, Ure and Wharfe. Sub-samples of juvenile Atlantic salmon (fry and parr stages) were euthanised with an overdose of buffered tricaine methanesulphonate (MS-222) and retained for analysis in the laboratory.

### Adult fish

Immigrating adult Atlantic salmon were captured from the Ouse, downstream of Naburn Weir (tidal limit; Fig. 1), between March and November 2021 by electrofishing from a boat. In addition, when no Atlantic salmon were apparent in the tidal Ouse, electric fishing was conducted immediately downstream of the next obstacle encountered by immigrating fish, namely Linton-on-Ouse Weir (Fig. 1). Eleven individuals were in suitable condition to be tagged (Supplementary Table 1). Fish were anaesthetised using MS-222 (0.8 g per 10 L of water), measured (fork length, millimetres) and weighed (grams), and fin clips were taken for genetic analysis. Acoustic transmitters (27.5 × 9 mm, 4.5 g weight in air (V9), 69 kHz; www.innovasea.com) were disinfected with povidone-iodine and rinsed with saline solution before being implanted into the body cavity through a small ventral incision, anterior to the pelvic fins. The incision was then closed with an absorbable monofilament suture and the fish allowed to recover in a large aerated, water-filled container treated with Virkon (0.5 g per 120 L; to reduce the risk of wound infection) and Vidalife (10 mL per 120 L; to aid wound healing). Fish were held and monitored until they had regained balance and began to actively swim before being released. Total transmitter weight in air did not exceed 2% of the body mass at the time of capture, to ensure it did not interfere with salmon behaviour or swimming performance. All fish were treated in compliance with the UK Animals (Scientific Procedures) Act 1986 (Home Office, project licence number PD6C17B56).

### Genetic analysis

#### DNA extraction, genotyping and quality control

DNA was extracted from fin samples by employing a modified modular universal DNA extraction protocol for tissue (Sellers et al. 2018) using a solid phase reversible immobilization (SPRI) magnetic bead capture method (adapted from Rohland and Reich 2012) to isolate high molecular weight DNA. Six individuals were included for cross-validation purposes. The DNA samples were sent to the Centre for Integrative Genetics (Ås, Norway) for genotyping. A custom 220,000-SNP Affymetrix Axiom array designed for Atlantic salmon (see Barson et al. 2015 for details) was used for data generation. Following the manufacturer’s instructions, only SNPs categorised as Poly High Resolution (high-confidence calls) and No Minor Homozygous (absence of minor allele homozygotes) were used in the analysis, while SNPs of unknown position were excluded from the dataset, leaving 214,632 loci available for genomic investigation. We then performed quality control (QC) and filtering of SNP data for the juvenile fish samples in PLINK versions (v.) 1.9 and 2.0 (www.cog-genomics.org/plink/1.9/ and www.cog-genomics.org/plink/2.0/; Purcell et al. 2007; Chang et al. 2015). SNPs were excluded from analysis if they failed to meet Hardy–Weinberg equilibrium (*P*-value < 0.001), had a minor allele frequency of < 5%, or exhibited > 10% missing genotype data. Individuals with more than 10% missing SNPs were also excluded. Given the polyandrous mating system of Atlantic salmon, PLINK 2.0 (–king-cutoff 0.0884) was used to identify second-degree and closer relatives (including half-siblings), retaining only one individual per family group to avoid potential confounding effects in downstream genetic analyses (Hansen et al. 1997). These QC steps resulted in the retention of 199,000 SNPs and 109 individual fish available for analysis from the rivers Swale (*n* = 30), Ure (*n* = 38) and Wharfe (*n* = 41). We also generated a linkage disequilibrium (LD)-pruned dataset in PLINK 1.9 (–indep 50 5 1.4), yielding 29,074 unlinked SNPs. The pruned dataset was used, where appropriate, to reduce the influence of LD in downstream structure and genome scan analyses; this is described in more detail in the following sections.

#### Genetic structuring and population diversity analyses

To investigate the genetic variation across tributaries in the Ouse catchment, a principal component analysis (PCA) was performed in PLINK 1.9 using the LD-pruned dataset of 29,074 unlinked SNPs from individuals in the rivers Swale, Ure and Wharfe. Using the same samples, ADMIXTURE v. 1.3. (Alexander et al. 2009) was used to infer the most likely number of genetic clusters (*K*; testing from *K* = 1 to *K* = 6), based on the lowest cross-validation error. The hierfstat v. 0.5.11 package (Goudet 2005) in R (R Core Team 2021) was used to calculatepairwise *F*_ST_ (Weir and Cockerham 1984) between tributary populations (genet.dist function, method = *WC84*), with 97.5% confidence intervals computed through bootstrapping (boot.ppfst function, 1000 permutations). Observed heterozygosity (*H*_o_) and gene diversity (*H*_s_) for each tributary were also calculated using the same package (basic.stats function), and compared using pairwise Wilcoxon tests. To assess genome-wide inbreeding, runs of homozygosity (ROH) were detected in PLINK v. 1.9 using conservative settings [minimum length 1 mega base (Mb), ≥ 100 SNPs per segment]. Individual genomic inbreeding coefficients were calculated as *F*_ROH_ = total ROH length divided by the total autosomal genome length covered by SNPs (McQuillan et al. 2008), estimated from SNP physical positions. Differences in *F*_ROH_ between rivers were assessed using a Kruskal–Wallis test followed by pairwise Wilcoxon tests. Finally, the current effective population size (*N*e) was calculated for each tributary in NeEstimator v. 2.1 (Do et al. 2014) using the unpruned 199,000 SNP dataset, thinned by physical distance to approximately one SNP every 50 kilo bases (kb) (resulting in 31,992 SNPs) to reduce computational load while retaining representative LD information. The LD method was used, with a lowest allele frequency threshold of 0.05. For this analysis, closely related individuals were retained (total number of individuals = 190; Swale = 57, Ure = 54, Wharfe = 79), as removing relatives can inflate LD-based estimates of *N*e (Waples 2025).

#### Genome scan for selection and gene annotation

We applied three complementary genome-scan approaches to detect SNPs showing unusually high differentiation between tributaries, as such loci may indicate genomic regions under selection and potential local adaptation. Results from the three methods were compared to identify SNPs consistently flagged as outliers, reducing the likelihood of false positives. Because ADMIXTURE analyses supported *K* = 2 and *K* = 3 as the most likely numbers of genetic clusters (see Results), we ran each genome scan by both (a) comparing the three tributary populations separately (*K* = 3), and (b) grouping the populations of the adjacent Swale and Ure into a single population and testing differentiation against the Wharfe population (*K* = 2).

First, we used the pcadapt v. 4.4.1 R package (Luu et al. 2017), which detects outlier loci based on their association with population structure, inferred from principal components. To reduce correlations between SNPs, analyses were conducted on the LD-pruned dataset. The resulting *P*-values were converted into *q*-values using the qvalue v. 2.30.0 R package (Storey et al. 2023). SNPs with *q*-values < 0.05 were considered candidate outliers, corresponding to an expected false discovery rate of 5%.

Second, we ran BayPass v. 2.3 (Gautier 2015), which estimates the *XtX* statistic, an *F*_ST_ analogue that accounts for covariance in allele frequencies between populations. To identify highly differentiated markers, we ranked SNPs by standardised *XtX* (*XtX*st) and considered the top 0.1% as candidate outliers.

Finally, we implemented the OutFLANK v. 0.2 R package (Whitlock and Lotterhos 2015), which is designed to detect loci with unusually high *F*_ST_ relative to the genome-wide background. It estimates a neutral distribution of *F*_ST_ from the majority of loci (after trimming the tails) and then identifies outliers that deviate significantly from this expectation. To avoid biases due to linkage, we fitted the neutral model on the LD-pruned dataset, but then applied the fitted parameters back to the full dataset. Loci with *q*-values < 0.05 were considered outliers.

To evaluate the potential functional roles of outlier genomic regions in local adaptation, for each method, we identified the ten nearest genes to every detected outlier SNP using the function closest in bedtools v. 2.29.1 (Quinlan and Hall 2010). From those ten, only genes within 10 kb upstream and downstream of outlier SNPs were retained to explore their potential role (Wellband et al. 2019). The *Salmo salar* Annotation Release 100 of the National Center for Biotechnology Information (ICSASG_v2) was used as the reference genome.

### Homing success rate

Natal homing behaviour and the accuracy of tagged Atlantic salmon were assessed using an array of 50 strategically located acoustic receivers (Vemco VR2W-69 kHz; www.innovasea.com) (Fig. 1; Supplementary Figure 1, Table 2). Specifically, three receivers were located at each major river confluence (i.e. Wharfe/Ouse, Nidd/Ouse, Swale/Ure) and downstream and upstream of potential migration barriers (Fig. 1). After the spawning season, the acoustic receivers were retrieved and any data downloaded. Individual fish movement routes and timing were analysed using the Actel R package v. 1.2.1 (Flávio and Baktoft 2021).

Homing accuracy was assessed by conducting a PCA on an expanded dataset incorporating both juveniles and adults to identify the tributary of origin for the immigrating adults. The same QC pipeline used for the main dataset was reapplied and resulted in 29,342 SNPs. If adults sampled in 2021 successfully migrated to and spawned in their natal river, we would expect adults and juveniles (sampled in 2019–2021) from the same tributary to cluster together in the PCA to the exclusion of the other tributaries. The telemetry data were then used to verify the findings, with the most upstream detection in the final tributary (if entered more than one) used to infer the spawning tributary of each fish. The number of adults returning to their natal tributaries was expressed as a percentage of the total number of tagged individuals.

### Geometric morphometric analysis

**Fig. 2:**
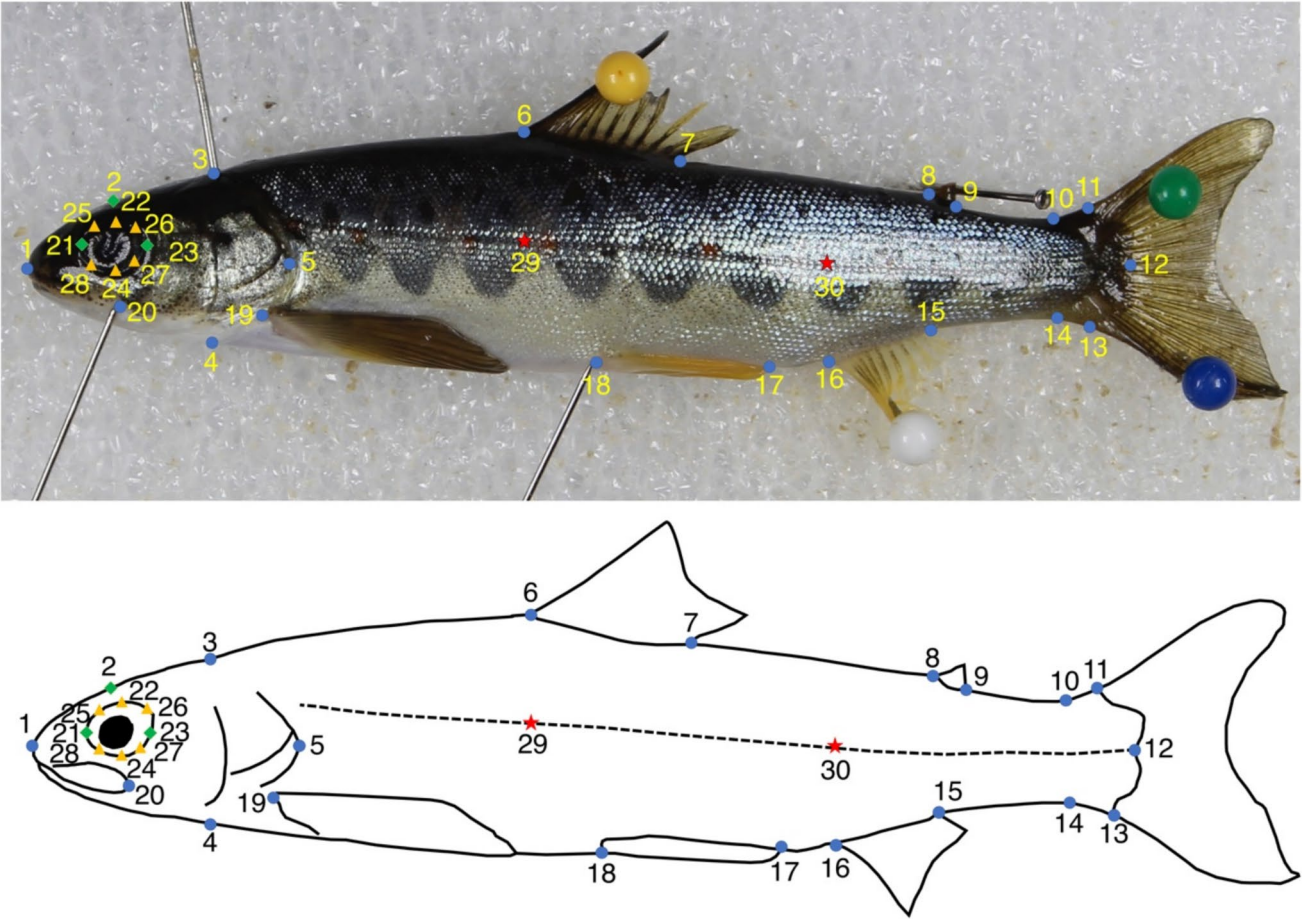
Fixed (*blue circles*) and semi-landmarks (*green diamonds*) and helper points (*orange triangles*) used for the geometric morphometric analyses of juvenile Atlantic salmon. The landmarks are projected onto a photograph of one of the samples (*upper figure part*) and are also shown in a drawing for easier visualization (*lower figure part*). Landmarks 29 and 30 (*red stars*) were used to correct for body arching and were not included in the analyses.*1* Tip of the snout;*2* midpoint between 1 and 3;*3* dorsal surface posterior to the cranium;*4* ventral surface posterior to the cranium;*5* posterior tip of the bony operculum;*6* anterior insertion point of the dorsal fin;*7* posterior insertion point of the dorsal fin;*8* anterior insertion point of the adipose fin;*9* posterior insertion point of the adipose fin;*10* dorsal insertion point of the caudal fin;*11* dorsal insertion point of the hypural plate;*12* posterior midpoint of the hypural plate;*13* ventral insertion point of the hypural plate;*14* ventral insertion point of the caudal fin;*15* posterior insertion point of the anal fin;*16* anterior insertion point of the anal fin;*17* posterior insertion point of the ventral fin;*18* anterior insertion point of the ventral fin;*19* anterior insertion point of the pectoral fin;*20* end of the maxillary bone;*21–28* eye outline;*29* lateral line, perpendicular to 6;*30* lateral line, perpendicular to 16. The figure was produced using Inkscape v. 0.92.2 (https://inkscape.org/)

The morphometric analysis was based on photographs of the genotyped individuals. All photographs were taken in the laboratory with a digital single-lens reflex camera (Canon EOS 4000D; focal length of 55 mm) fixed on a copy stand illuminated by two light-emitting diodes. The fish were then sexed by inspecting the gonads, and precocious males (*n* = 18) were excluded as preliminary analysis showed they had a markedly different body shape, which would have biased the analysis. As a result, 145 individuals from the rivers Swale (*n* = 42), Ure (*n* = 45) and Wharfe (*n* = 58) were analysed. Thirty points, namely 19 fixed, three semi-landmarks and eight helper points, were digitised by a single operator, using tpsDig v. 1.47 (Fig. 2; Rohlf 2019), and the coordinates imported into R and analysed using geomorph and RRPP v. 3.2.0 (Adams et al. 2021; Baken et al. 2021; Collyer and Adams 2021), Morpho v. 2.8 (Schlager 2017), and GeometricMorphometricsMix v. 0.0.8.4 (Fruciano 2018) packages. Figures and plots were produced with the geomorph and ggplot2 packages (Wickham 2016).

Preliminary analysis revealed body bending as a major source of shape variation in the dataset. This was corrected in tpsUtil v. 1.78 (Rohlf 2017) by employing landmarks 1, 29, 30 and 12 (see Moccetti et al. 2023 for details). Landmarks 29 and 30 were then discarded from the analysis. First, a generalised Procrustes analysis (GPA) was performed to remove effects not related to body shape through translation, scaling and rotation of the landmark configurations (Rohlf and Slice 1990). The minimum Procrustes distance criterion was used for sliding of semi-landmarks (Perez et al. 2006). Helper points around the eye were treated as sliding semi-landmarks in the GPA, to facilitate the alignment of the two eye semi-landmarks (points 21 and 23; Fig. 2), and were subsequently removed from the dataset (Raffini et al. 2018; Fruciano et al. 2020). All the following analyses were based on 22 landmarks.

The effect of sex on body shape was tested using Procrustes ANOVAs, with Procrustes coordinates used as an outcome variable, Sex and River and their interaction as independent variables and a randomised residual permutation procedure (10,000 iterations). If the interaction term Sex:River was found to be statistically significant, the females and males were analysed separately. First, a GPA was computed on landmark coordinates, then the residual effect of fish size on body shape was tested using Procrustes ANOVAs, with Procrustes coordinates used as an outcome variable, and the log value of centroid size and River and their interaction as independent variables (10,000 permutations). If an effect of size on shape was found, then Procrustes coordinates were adjusted for allometry by using residuals from a regression of shape against centroid size + River. Procrustes ANOVAs were then used to compare mean body shape between tributaries. A between-group PCA (Boulesteix 2004) was computed in Morpho to explore variations between the three tributaries. The leave-one-out cross-validation, an option within the between-group PCA in Morpho, was implemented to quantify the proportion of fish correctly assigned to their tributary of origin.

## Results

### Genetic structuring and population diversity

The sibship analysis identified and removed several putative second-degree and closer relatives from the datasets of the three tributaries (Ure, *n* = 16; Swale, *n* = 27; Wharfe, *n* = 35).

**Fig. 3a–c:**
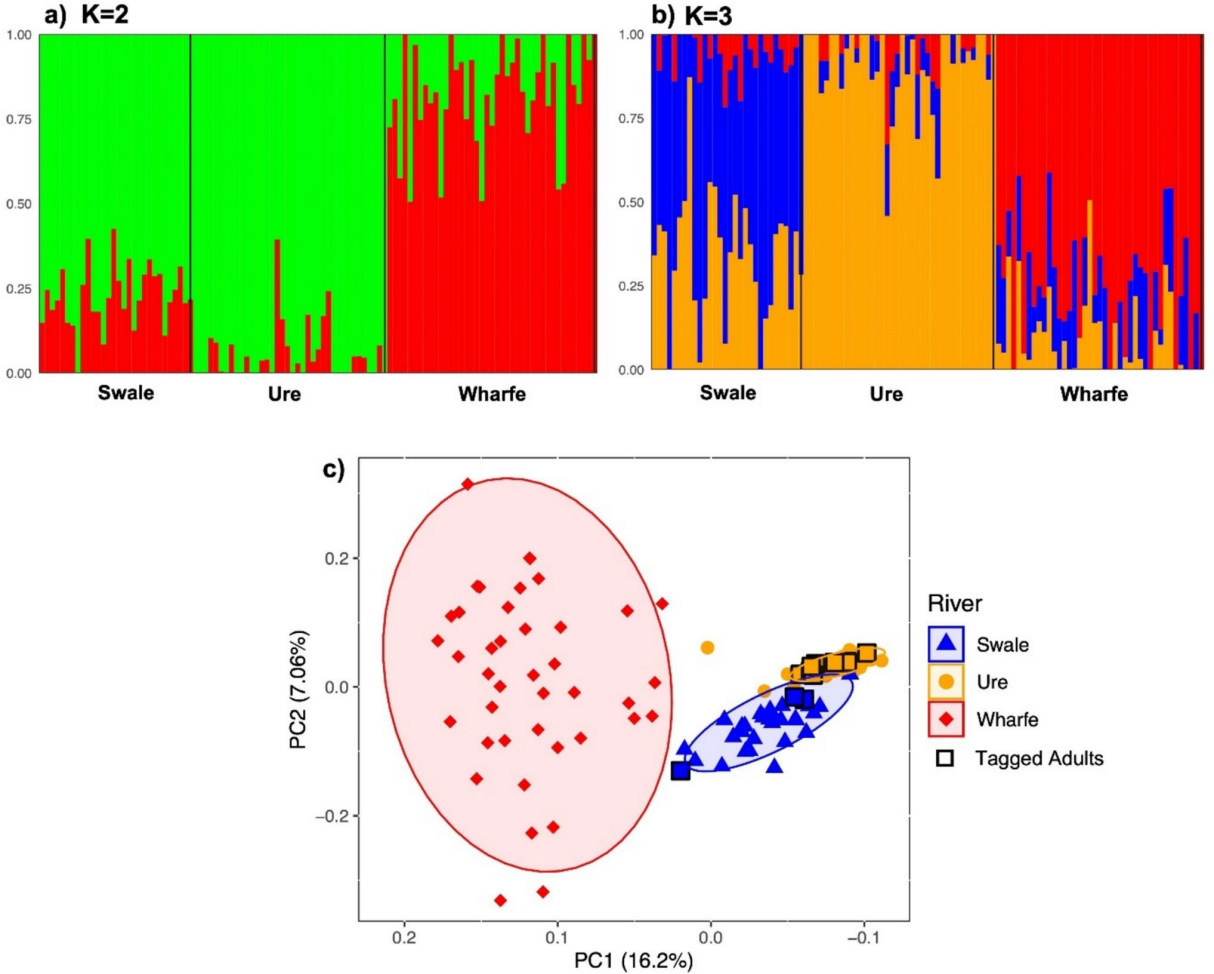
Genetic structuring of Atlantic salmon populations in the Yorkshire Ouse tributaries. **a**, **b** ADMIXTURE plots for *K* = 2 and *K* = 3. At *K* = 2 (**a**), fish from the Ure and Swale form a single genetic group distinct from that from the Wharfe, whereas at *K* = 3 (**b**) the clusters correspond to the populations from the three tributaries. Each*vertical bar* represents an individual juvenile fish. **c** Principal component analysis (PCA) scatterplot, where*each symbol* represents an individual fish.*Open squares* represent adult fish tracked for homing accuracy. Adults are*coloured* according to their spawning tributary, as identified through telemetry. The variance (%) explained by the first and second axes is shown

ADMIXTURE analysis identified two genetic clusters (*K* = 2; cross-validation error = 0.55133; Supplementary Figure 2) as the most likely grouping, closely followed by three clusters (*K* = 3; cross-validation error = 0.55561, Supplementary Figure 2). Under the *K* = 2 scenario, fish from the Ure and Swale formed a single genetic group that was distinct from the Wharfe (Fig. 3a). Under the *K* = 3 assignment, the clusters corresponded to the three tributaries, with a partial admixing of individuals from the Swale and Ure (Fig. 3b). The PCA successfully separated fish from the Swale, Ure and Wharfe, with those from the Ure and Swale forming closer clusters along PC1 (Fig. 3c; Supplementary Figure 3). The pattern of greater similarity between the populations of the geographically adjacent Ure and Swale was supported by the significantly (i.e. non-overlapping confidence intervals, Supplementary Table 3) lower pairwise genetic distance between the Swale and Ure (*F*_ST_ = 0.015) compared with Swale–Wharfe (*F*_ST_ = 0.031) and Ure–Wharfe (*F*_ST_ = 0.042).

The population statistics showed that the Ure had the lowest genetic diversity (*H*_o_ and *H*_s_), followed by the Swale and Wharfe (*P*-values < 0.0001; Table 1), which was consistent with the observed cluster sizes in the PCA (Fig. 3c). Genomic inbreeding estimates based on *F*_ROH_ supported this pattern, with the Ure displaying the highest *F*_ROH_ (0.064; *P*-values < 0.0001), while the estimates for the Swale and Wharfe did not differ significantly (*P*-value = 0.46; Table 1). In contrast, estimates of *N*e indicated the opposite pattern, with the Ure showing the largest *N*e (≈ 79), followed by the Wharfe (*N*e ≈ 46) and then the Swale (*N*e ≈ 32; Table 1).

**Table 1:** Estimates of observed heterozygosity (*H*_*o*_), gene diversity (*H*_*s*_), genomic inbreeding based on runs of homozygosity (*F*_*ROH*_; median with interquartile range), and effective population size (*N*e; with 95% jackknife confidence intervals) for juvenile Atlantic salmon from each Yorkshire Ouse tributary

River	*N*	*N*(*N*e)	*H*_o_	*H*_s_	*F*_ROH_	*N*e
Swale	30	57	0.340	0.332	0.029 (0.019–0.067)	32.2 (20.6–55.3)
Ure	38	54	0.320	0.311	0.064 (0.057–0.092)	78.9 (43.0–234.0)
Wharfe	41	79	0.350	0.344	0.027 (0.015–0.050)	45.7 (30.6–74.1)

*N* Number of individuals used for diversity and ROH analyses, *N*(*N*e) number of individuals used for *N*e estimation

### Genome scan for selection

**Fig. 4a, b:**
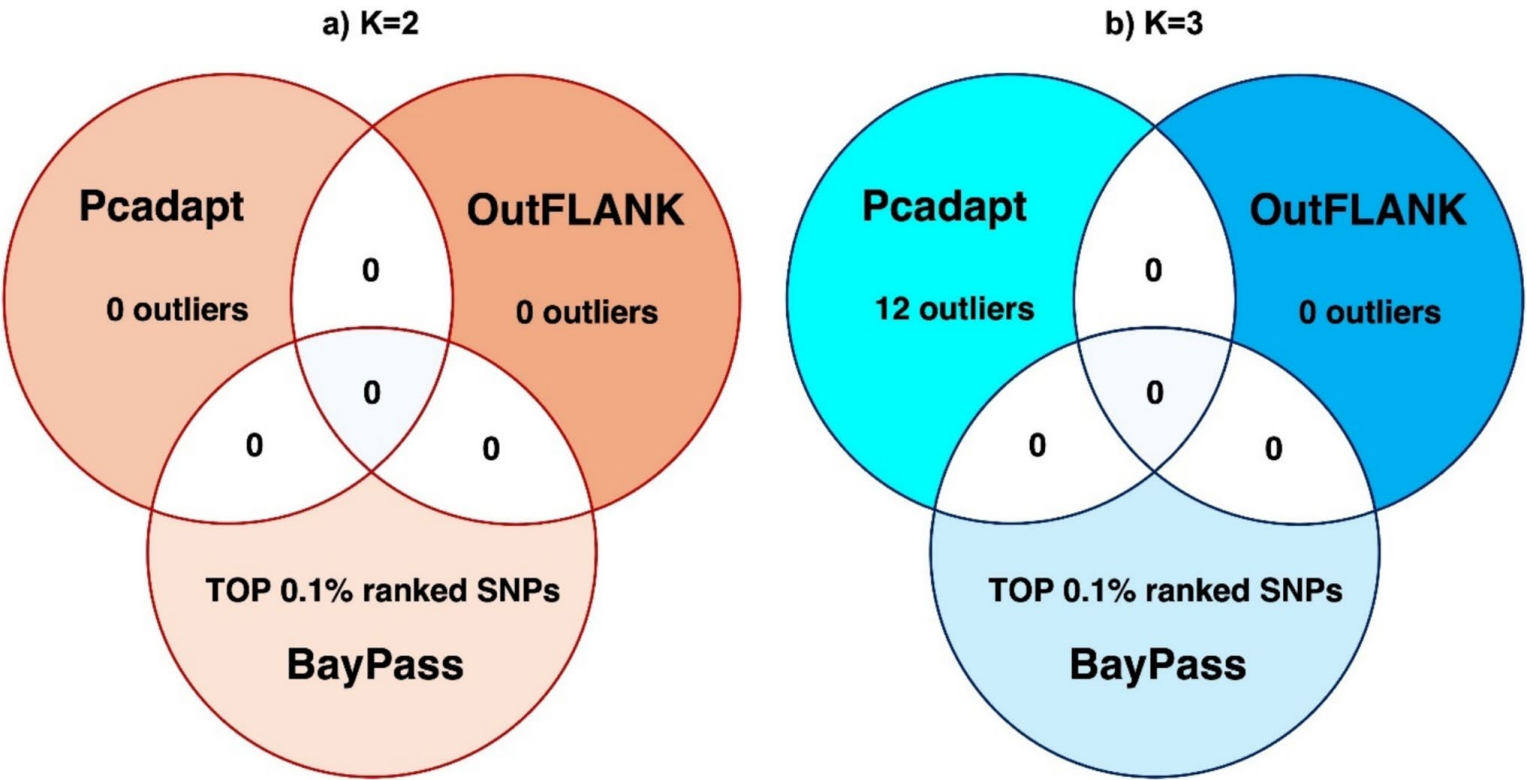
Venn diagrams summarising results from outlier analyses performed with pcadapt, OutFLANK, and BayPass to detect putative signals of selection between recovering Atlantic salmon populations in the Yorkshire Ouse. **a** *K* = 2 scenario, contrasting the Wharfe group with the combined Ure and Swale group. **b** *K* = 3 scenario, treating the Swale, Ure, and Wharfe groups as three separate populations. No loci were identified as outliers by more than one method, and thus no overlaps are present.*SNP* Single nucleotide polymorphism

Outlier detection analyses revealed limited signals of selection under both the *K* = 2 and *K* = 3 grouping schemes (Fig. 4a, b). Under the *K* = 2 scenario (Fig. 4a), neither OutFLANK nor pcadapt identified any candidate outlier SNPs, and therefore no cross-validation with the top 0.1% *XtX* candidate SNPs and nearby genes from BayPass (199 SNPs; Supplementary Files 1, 2) was possible. Under the *K* = 3 (three-tributary) dataset (Fig. 4b), OutFLANK again detected no candidate loci, whereas pcadapt identified 12 outlier SNPs located within ± 10 kb of ten genes (Supplementary Figure 4; Supplementary Files 3, 4). However, none of these pcadapt outliers, nor their nearby annotated genes, overlapped with the top 0.1% BayPass SNPs or the genes located near them (Supplementary Files 5, 6).

### Homing success rate

**Fig. 5a–c:**
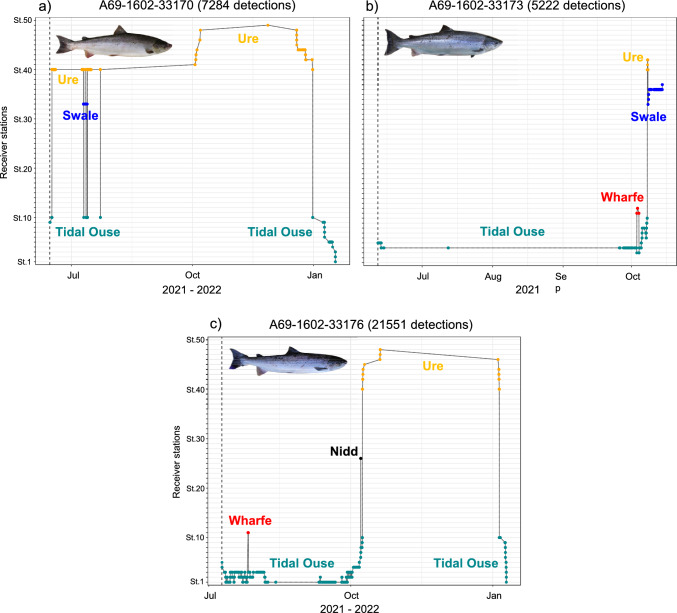
Migration paths of three immigrating Atlantic salmon displaying possible exploratory behaviour before homing to their natal river, as predicted by genetic analyses. **a**, **c** These fish emigrated after spawning. The *y-axis* represents the locations where the fish were detected. Due to the dendritic nature of the study area, the sequential numbering of the stations may not consistently reflect their order upstream as encountered by the fish (see Supplementary Figure 1, Table 2)

Adult Atlantic salmon exhibited a range of migratory tactics, but none migrated to the spawning grounds immediately after release (Supplementary Figure 5). Seven fish spawned in the Ure, three spawned in the Swale, one may have spawned in the Wharfe, and none spawned in the Nidd (Supplementary Figure 5). The overall homing accuracy was estimated to be approximately 91%, since all but one of the tagged fish migrated to their river of origin, based on genetic structure analysis (Fig. 3c; Supplementary Figures 6, 7). The straying fish belonged to the Ure genetic cluster and undertook an early season movement to the Wharfe, but ceased to be detected in mid-September, before the onset of the spawning season (Supplementary Figures 5g, 6). While most of the fish migrated directly to their natal tributary, three exhibited possible exploratory behaviours, entering various tributaries before continuing to their spawning destination (Fig. 5). One individual entered the Swale before spawning in the Ure (Fig. 5a), one entered the Wharfe and Ure before spawning in the Swale (Fig. 5b), and one entered the Wharfe and Nidd before spawning in the Ure (Fig. 5c). All three of these fish eventually moved to their natal river, as predicted by the genetic structuring analysis (Fig. 3c, Supplementary Figure 7).

### Geometric morphometric analysis

The interaction term Sex:River was found to be statistically significant (*P*-value = 0.0025, *r*^2^ = 0.033), indicating that the effect of Sex on shape varied significantly across different rivers. Females and males were therefore analysed as separate subsets. No effect of size on shape was found for males, whereas a small but significant effect was found in all tributaries for females (*P*-value = 0.0146, *r*^2^ = 0.049). Procrustes coordinates were therefore adjusted for allometry in the latter dataset.

**Fig. 6:**
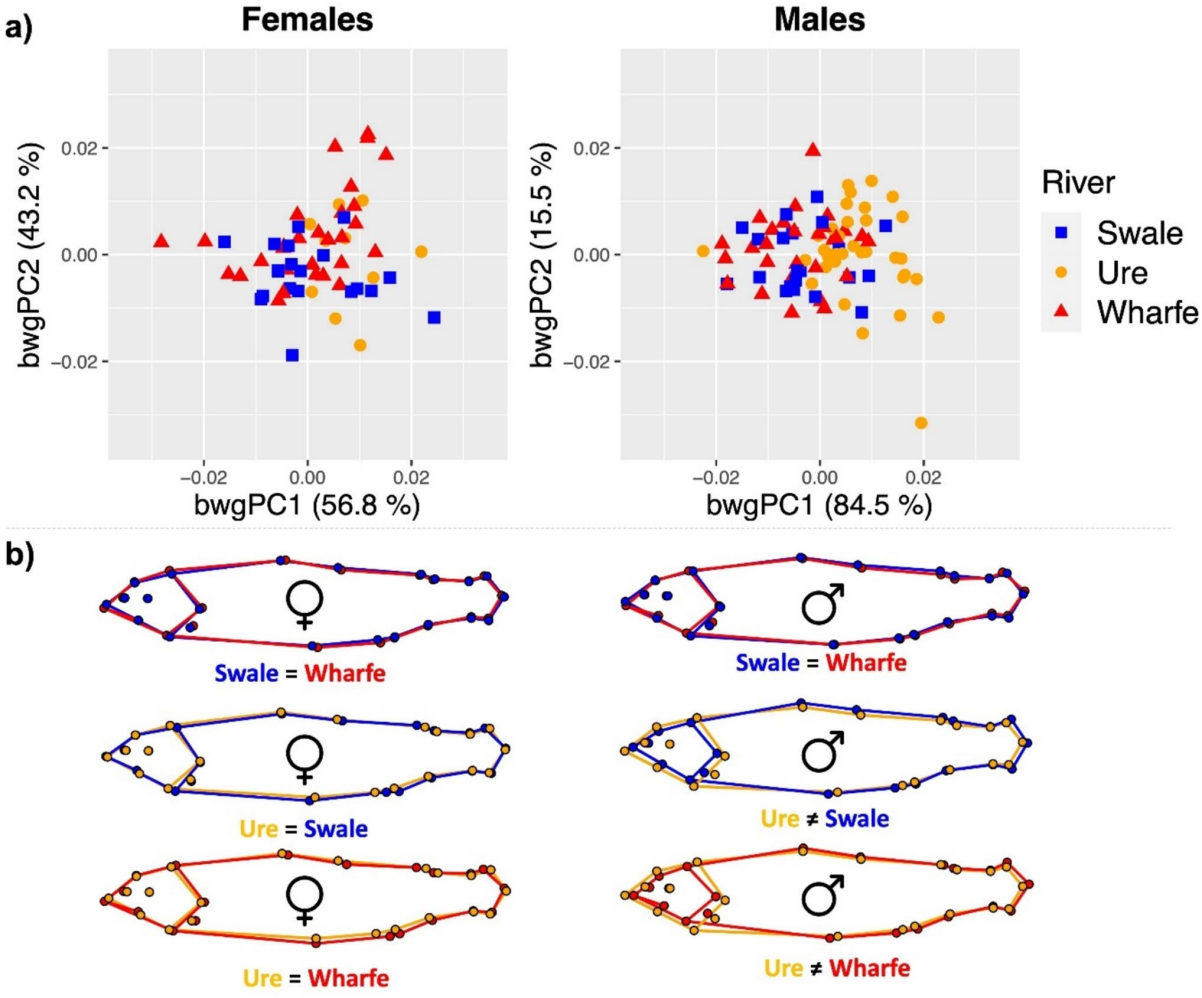
Between-group (*bwg*) PCA scatterplots (**a**) and mean body shape projections (**b**) showing morphological comparisons between Yorkshire Ouse tributaries for female and male juvenile Atlantic salmon. In the PCA plot (**a**),*dots* represent individual fish, and the variance (%) explained by the first and second bwg PCs is shown. In the shape projections (**b**), the*equal symbol* (=) indicates no significant difference (*P*-value ≥ 0.05) while the not equal symbol (≠) indicates a significant difference in shape (see Supplementary Table 4 for *P*-values and Euclidean distances). Morphological differences in the projections were magnified 3 times to aid visualisation

There were no differences in body shape between females from different tributaries (*P*-value = 0.5093; Table 2; Fig. 6a). In contrast, males from the Ure had a significantly different morphology compared to those from the rivers Swale and Wharfe (*P*-value = 0.001; Table 2; Fig. 5a; Supplementary Table 4). These variations were concentrated in the head region, with males from the Ure displaying a larger head and wider eye (Fig. 6b).

**Table 2:** Procrustes ANOVA summary statistics of the effect of river on the body shape of female and male juvenile Atlantic salmon

Dataset	*df*	SS	*r*^2^	*F*	*Z*	*P*-value
Females	2	0.001	0.031	0.903	− 0.035	0.5093
Males	2	0.004	0.086	3.864	4.366	0.0001

SS Sum of squares, *Z* effect size

## Discussion

This study revealed significant genetic structuring in Atlantic salmon within the Yorkshire Ouse catchment, consistent with reproductive isolation between the populations of tributaries maintained by philopatry (homing behaviour). However, morphological variation between the populations of the tributaries was negligible and no clear genomic signal of local adaptation was found. A clear hierarchical genetic structure was evident, with the Wharfe cluster separating from the combined Ure-Swale cluster at *K* = 2 (ADMIXTURE analysis), and populations of all three tributaries differentiating at *K* = 3, reflecting the geographic distance of these watercourses within the catchment. The genetic distances (*F*_ST_) between the Atlantic salmon of these tributaries were comparable to those observed using the same SNP chip between long-established Atlantic salmon populations from distinct river systems (Pritchard et al. 2018; Zueva et al. 2021).

Despite this overall genetic differentiation, we detected no evidence of specific genomic regions showing signals of diverging selection between the populations of these rivers (i.e. no putatively adaptive loci). Outlier detection methods yielded inconsistent results: very few candidates were identified and there was no overlap across approaches for either *K* = 2 or *K* = 3. Together, these patterns suggest that the identified candidates are likely false positives, a common outcome in outlier analyses (Luu et al. 2017), and too weak to represent robust evidence of divergent selection, especially when compared with the patterns reported in other salmonid studies that typically show strongly differentiated loci (Waples et al. 2020). Similarly, body shape, a key fitness-related trait that can respond rapidly to environmental conditions, even within an individual’s lifetime, as a plastic response (Adams et al. 2003), was largely homogeneous across tributaries. This suggests similar selective pressures and environmental conditions across the three tributaries, which are characterised by comparable animal and plant communities, flow regimes, and geology (Jarvie et al. 1997; Law et al. 1997; Tipping et al. 1997; Whitton and Lucas 1997). Altogether, these findings suggest that the observed genetic differentiation was primarily driven by random demographic processes (such as genetic drift and historical population changes) rather than divergent selection or local adaptation.

This latter conclusion is consistent with the low contemporary *N*e determined for all three tributaries, with only that of the Ure exceeding the commonly cited short‐term threshold of *N*e ≥ 50 for the avoidance of severe inbreeding depression in closed populations (Franklin 1980). At such small *N*e values, genetic drift is expected to be strong, with random changes in allele frequencies between generations potentially playing a major role in shaping current patterns of genetic differentiation (Waples 2025). While these small population sizes are consistent with a recovery scenario driven by a limited number of individuals (i.e. a founder effect following a severe genetic bottleneck), estimates of genetic diversity based on *F*_ROH_, *H*_o_, and *H*_s_ did not indicate severe inbreeding or critically low diversity. *F*_ROH_ values were moderate and comparable to those reported for other semi‐isolated wild salmonid populations (Tengstedt et al. 2024; Saha et al. 2024; Bell et al. 2025), while *H*_o_ and *H*_s_ values were consistent with those observed in long‐established Atlantic salmon populations (Cauwelier et al. 2018; Pritchard et al. 2018; Zueva et al. 2021). Notably, similar patterns were reported by Jensen et al. (2017), detecting analogous heterozygosity values (0.332–0.352) in Atlantic salmon that colonised two newly available river branches and diverged in fewer than 15 generations. This paradox of relatively high genetic diversity despite population bottleneck due to colonisation or demographic decline (Maduna et al. 2024) may be attributed to the minimal impact of low-frequency alleles on overall heterozygosity and inbreeding, making potential reductions in diversity less detectable (Allendorf et al. 2024).

Inferring the specific eco-evolutionary processes that followed the historical population bottleneck in the Ouse and led to the observed genetic structure is not straightforward, as historical stocking from both exogenous and indigenous sources, natural recolonisation from other rivers, and the possible persistence of remnant individuals from original populations may all have contributed to this. The stocking of fish from Scotland (between 1965 and 1975) (Axford 1991) could have been a plausible explanation for the observed structuring if genetically distinct fish had been introduced into the three Ouse tributaries; however, that was not the case, as only the Ure had been stocked. Furthermore, Atlantic salmon in the Ure are genetically distinct from all major Scottish populations, including those from which fish were stocked in the 1960s and 1970s (Gilbey et al. 2016). This distinction is also supported by findings from other work that used the same SNP chip and the Ure samples employed in this study (Moccetti et al. 2024). All of this, combined with the poor return rates of the hatchery-reared fish (Howes 1976; Axford 1991), suggests that stocking with Scottish fish did not substantially affect the Atlantic salmon in the Ouse.

In light of this, there are two plausible explanations for the observed population structure: Atlantic salmon recolonised the area from elsewhere, and/or salmon persisted within the Ouse system. The first hypothesis is consistent with reports of natural recolonisation of river systems by Atlantic salmon from other populations (Vasemägi et al. 2001; Grandjean et al. 2009; Perrier et al. 2009; Griffiths et al. 2011; Ikediashi et al. 2012). Within this context, multiple waves of colonisation into different tributaries, followed by homing fidelity, could explain the observed genetic structuring in the Ouse. Alternatively, rapid genetic differentiation following a single colonisation event could have occurred locally within a period of approximately 40 years since Atlantic salmon reappeared in the system (i.e. eight generations), as has been reported in other salmonid systems (Hendry et al. 2000; Jensen et al. 2017; Sparks et al. 2023). However, research has shown that fish from the Ouse tributaries are genetically distinct from those of all major Scottish populations (Gilbey et al. 2016; Moccetti et al. 2024), which could otherwise represent likely sources of the fish given the short geographic distance and high population abundance, and therefore higher potential for straying. In the absence of baseline genetic data from other nearby English populations and more distant rivers, it remains difficult to conclusively identify donor populations or evaluate their relative contributions.

The second hypothesis, that the genetic differentiation is a result of isolation over a long period of time, implies that, contrary to circumstantial and anecdotal evidence (Howes 1976; Axford 1991; Lucas et al. 1998), Atlantic salmon were never completely extirpated from the catchment, with the original populations persisting, despite substantial historical reductions in size. This phenomenon has been documented in Danish rivers, where genetic studies revealed that some individuals from the original populations, previously thought to have been fully extirpated, were still present, thus demonstrating an unexpected resilience, even if extensive stocking with hatchery fish of exogenous origin had occurred (Nielsen et al. 1997; Koed et al. 2020). Unfortunately, genetic samples from before the putative extinction of Atlantic salmon in the Ouse are not available, preventing the direct testing of this hypothesis. Thus, disentangling the relative contributions of these processes to the current genetic structure remains challenging, since remnant populations, recolonisation, and recent local differentiation may all have played simultaneous roles in it.

Regardless of the specific processes involved, the observed sub-catchment genetic differentiation has occurred without physical barriers to gene flow. Although within-catchment structuring in Atlantic salmon is known to occur (Vaha et al. 2007; Ensing et al. 2011; Perrier et al. 2014; Miettinen et al. 2021), it is not universally observed, and its underlying drivers are not fully understood (Dionne et al. 2009; Wellband et al. 2019). Colonisation history, geomorphology and ecological dynamics likely interact in this intricate process (Dillane et al. 2008). However, the key role played by homing behaviour in the promotion and maintenance of reproductive isolation and, thus, genetic divergence is widely recognised (Garcia De Leaniz et al. 2007). It has been hypothesised that, when rivers exhibit similar or non-stable environmental characteristics over time and/or are geographically proximate, homing may be less accurate and, consequently, genetic structuring weaker (Dittman and Quinn 1996; Cauwelier et al. 2018). However, here, we demonstrate the ability of fish to actively discriminate between tributaries, such as the Swale and Ure, which are situated just a few kilometres apart from their sources to the confluence and seem to exhibit similar ecological and environmental conditions (Jarvie et al. 1997; Law et al. 1997; Tipping et al. 1997; Whitton and Lucas 1997). Furthermore, three fish were detected moving among different tributaries, including the confluent Swale and Ure, before spawning in their natal river. Although such behaviour has been documented in Atlantic salmon (Thorstad et al. 2008) and other salmonid species (Keefer and Caudill 2014), the origins of the fish were unknown, and determining whether the spawning location was in their natal river was not possible. To our knowledge, this study provides the first preliminary evidence of Atlantic salmon displaying “search” behaviour before eventually homing to their natal river, as observed for sockeye salmon (*Oncorhynchus nerka* Walbaum, 1792) (Peterson et al. 2016). This novel result underscores the importance and potential of combining genetic and telemetry tools in studies on fish movement (Östergren et al. 2012; Moore et al. 2017).

Notwithstanding the findings discussed above, there was some evidence of limited admixture between the Swale and Ure populations. While Ure fish appear genetically distinct, some individuals from the Swale show admixture with the Ure, suggesting asymmetric gene flow. One possible explanation for this is that the Swale was recolonised recently by straying Ure fish, as supported by historical survey records indicating an absence of salmon in the Swale during the 1990s and their reappearance in small numbers in the 2000s (Environment Agency 2024). This recolonisation may have been facilitated by growing Atlantic salmon numbers in the neighbouring Ure, combined with improvements in water and habitat quality in the area (Neal and Robson 2000). Alternatively, the observed admixture might be linked to the stocking of the Ure with local broodstock in the 2010s, as hatchery-reared fish typically exhibit reduced homing accuracy and may stray into nearby tributaries (Jonsson et al. 2003; Salmenkova 2017). However, further research is required to directly test this possibility. The recent stocking of the Ure may also help explain why this tributary showed the lowest genetic diversity (lowest *H*_o_ and highest *F*_ROH_), while simultaneously exhibiting the highest *N*e. Reduced genetic diversity is a well-documented consequence of stocking with progeny from a limited number of brood fish (the Ryman-Laikre effect) (Ryman and Laikre 1991; Jasper et al. 2013), whereas the relatively high *N*e found here likely reflects a short-term increase in census size and the number of breeding individuals following supplementation and subsequent population growth. Although current levels of inbreeding in the Ure appear to be low to moderate, these patterns highlight the importance of continued genetic monitoring to ensure the long-term viability and adaptive potential of the population.

## Conclusions

Overall, this study reveals distinct genetic structuring between Atlantic salmon in the recovering Ouse system, which is maintained by accurate homing behaviour. The patterns are best explained by neutral demographic processes rather than divergent selection, consistent with the absence of adaptive genomic or morphological differentiation. These findings have significant implications for conservation, particularly in the current period of declining population sizes and ranges of many diadromous fish species, as they highlight how improved environmental conditions can support the recovery of remnant populations and/or facilitate recolonisation, thus contributing to genetic differentiation through homing, and potentially the formation of independent demographic and evolutionary units. Although no evidence of adaptive divergence was detected in the present study, this does not preclude the emergence of local adaptation in the near-term, since rapid adaptive changes have been documented in salmonids over short ecological timescales (Haugen et al. 2008; Czorlich et al. 2022), including in populations founded by a limited number of individuals (Sparks et al. 2024). Future research that integrates genomic and diverse phenotypic data across different life stages is essential to increase our understanding of the pace and process of local adaptation during the recovering process of diadromous fish populations. Continued genomic and ecological monitoring of recovering populations, such as those in the Yorkshire Ouse catchment, are crucial for detecting these dynamics over time and for informing conservation strategies for Atlantic salmon and other threatened species.

## Supplementary Information

Below is the link to the electronic supplementary material.

Supplementary file1 (PDF 2116 KB)

Supplementary file2 (CSV 20 KB)

Supplementary file3 (CSV 23 KB)

Supplementary file4 (CSV 1 KB)

Supplementary file5 (CSV 1 KB)

Supplementary file6 (CSV 19 KB)

Supplementary file7 (CSV 24 KB)

## Data Availability

The code used for the analyses is publicly available at https://tinyurl.com/Yorkshire-Salmon.
