# Supplementary material for: Genomic, morphological and migratory patterns in recovering Atlantic salmon populations
Source: Aquat Sci. 2026 Mar 30;88(2):65. doi: 10.1007/s00027-026-01288-1 (PMC13098855; doi:10.1007/s00027-026-01288-1)
Supplement: Supplementary file 1 — Supplementary file1 (PDF 2116 KB) [file 27_2026_1288_MOESM1_ESM.pdf]

Supplementary Material for:

Genomic, morphological and migratory patterns in recovering Atlantic salmon populations (Moccetti et al. 2025).

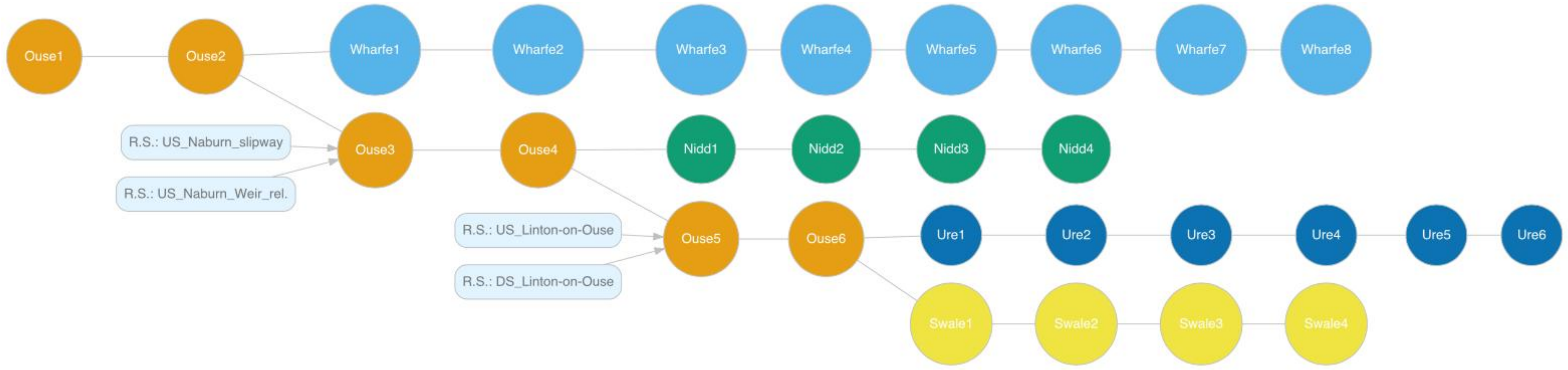

Supplementary Figure 1. Schematic representation of the potential routes taken by immigrating Atlantic salmon in the Yorkshire Ouse catchment.

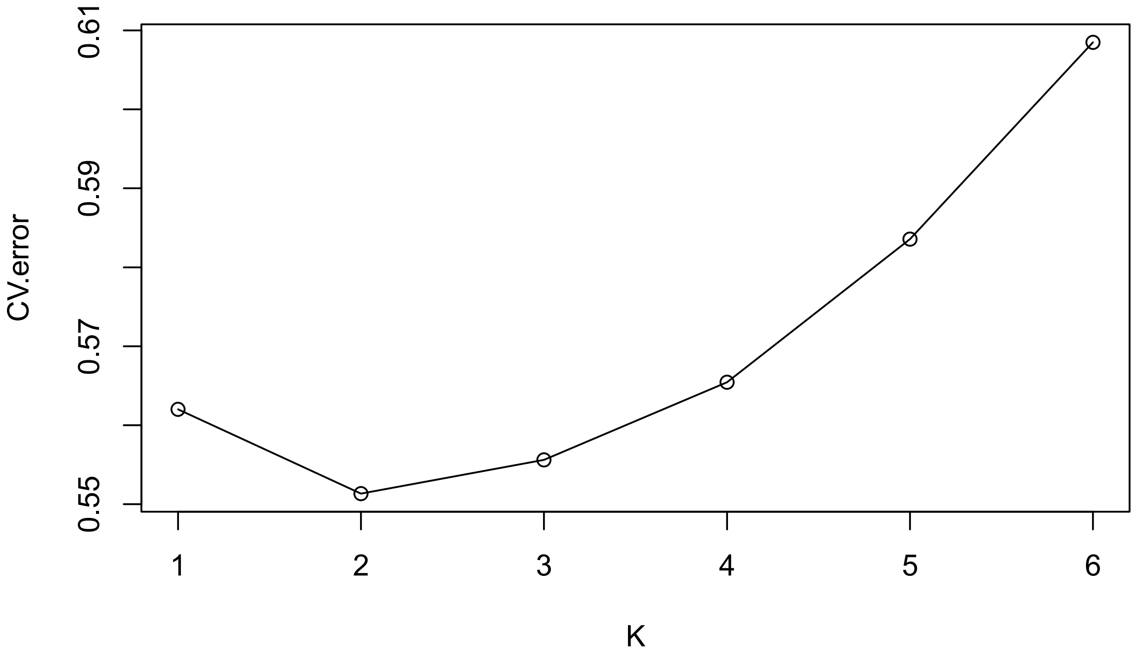

Supplementary Figure 2. Cross-validation error (x-axis) for each K (number of genetic units) tested by ADMIXTURE. The most likely number of genetic clusters (i.e. K = 2 or K = 3) was inferred based on the lowest cross-validation error.

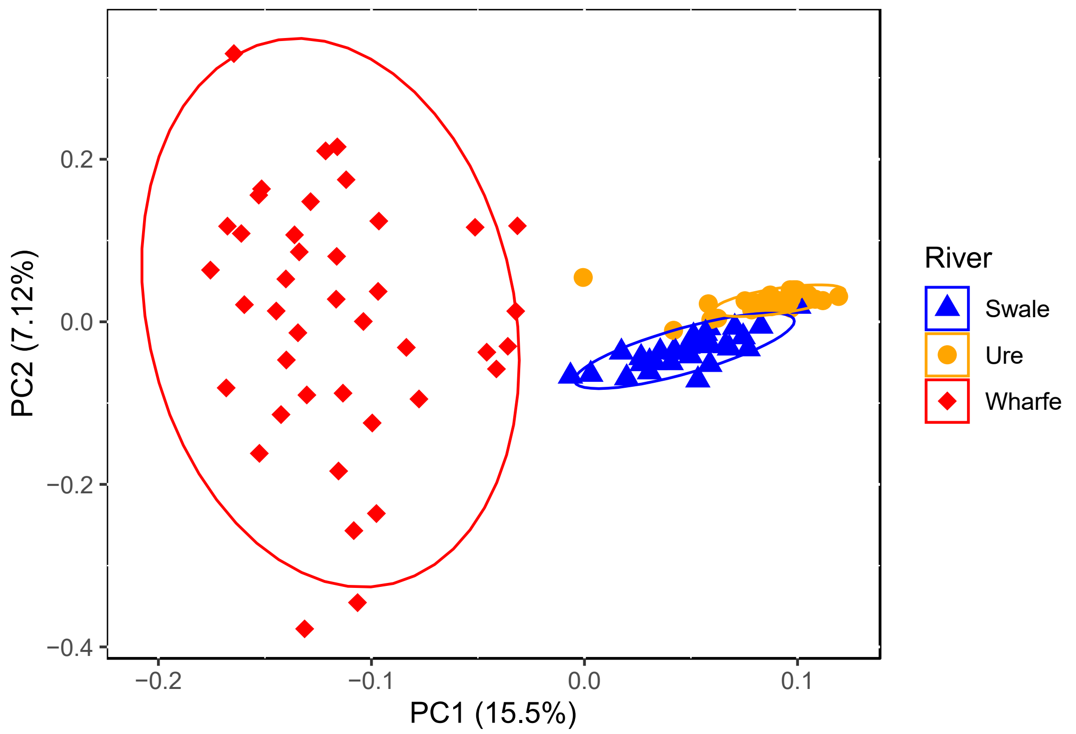

Supplementary Figure 3. Genetic structuring of Atlantic salmon populations in the Yorkshire Ouse tributaries based on 29,074 SNPs. In the PCA scatterplot dots represent individual juvenile fish and the variance (%) explained by the first and second axes is shown. Colours correspond to tributaries.

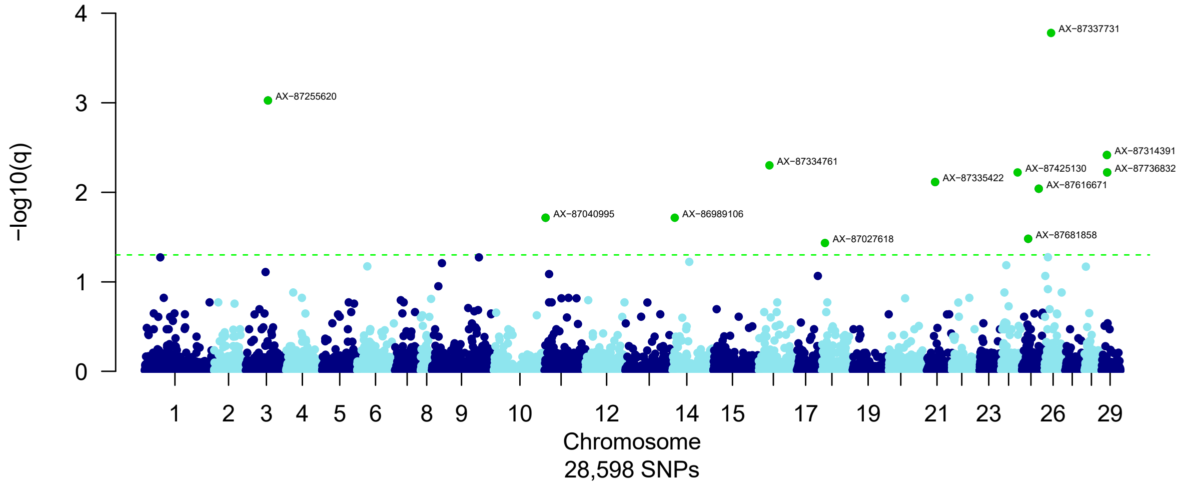

Supplementary Figure 4. Manhattan plot showing genome-wide q-values from the pcadapt analysis under the K = 3 scenario. Each point represents a SNP, plotted according to its genomic position (x-axis) and -$\log_{10}$(q) value (y-axis). The dashed green line indicates the q = 0.05 threshold. Candidate outlier SNPs ($q < 0.05$) are highlighted in green and annotated with their marker IDs. Chromosome numbers and the total number of SNPs analysed (28,598) are shown along the x-axis.

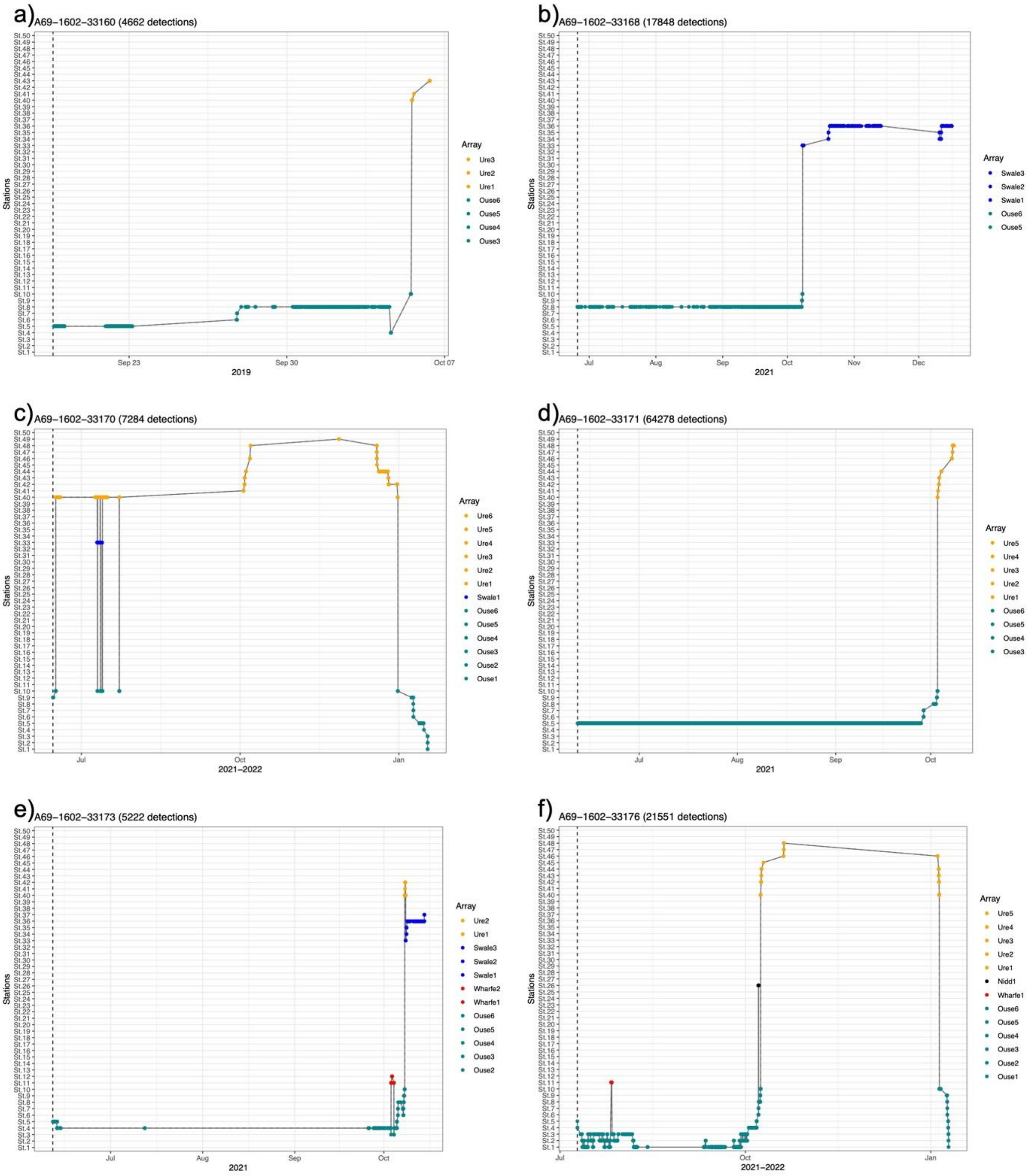

a) A69−1602−33160 (4662 detections)
Stations
2019
Sep 23
Sep 30
Oct 07
Array
Ure3
Ure2
Ure1
Ouse6
Ouse5
Ouse4
Ouse3
b) A69−1602−33168 (17848 detections)
Stations
2021
Jul
Aug
Sep
Oct
Nov
Dec
Array
Swale3
Swale2
Swale1
Ouse6
Ouse5
c) A69−1602−33170 (7284 detections)
Stations
2021−2022
Jul
Oct
Jan
Array
Ure6
Ure5
Ure4
Ure3
Ure2
Ure1
Swale1
Ouse6
Ouse5
Ouse4
Ouse3
Ouse2
Ouse1
d) A69−1602−33171 (64278 detections)
Stations
2021
Jul
Aug
Sep
Oct
Array
Ure5
Ure4
Ure3
Ure2
Ure1
Ouse6
Ouse5
Ouse4
Ouse3
e) A69−1602−33173 (5222 detections)
Stations
2021
Jul
Aug
Sep
Oct
Array
Ure2
Ure1
Swale3
Swale2
Swale1
Wharfe2
Wharfe1
Ouse6
Ouse5
Ouse4
Ouse3
Ouse2
f) A69−1602−33176 (21551 detections)
Stations
2021−2022
Jul
Oct
Jan
Array
Ure5
Ure4
Ure3
Ure2
Ure1
Nidd1
Wharfe1
Ouse6
Ouse5
Ouse4
Ouse3
Ouse2
Ouse1

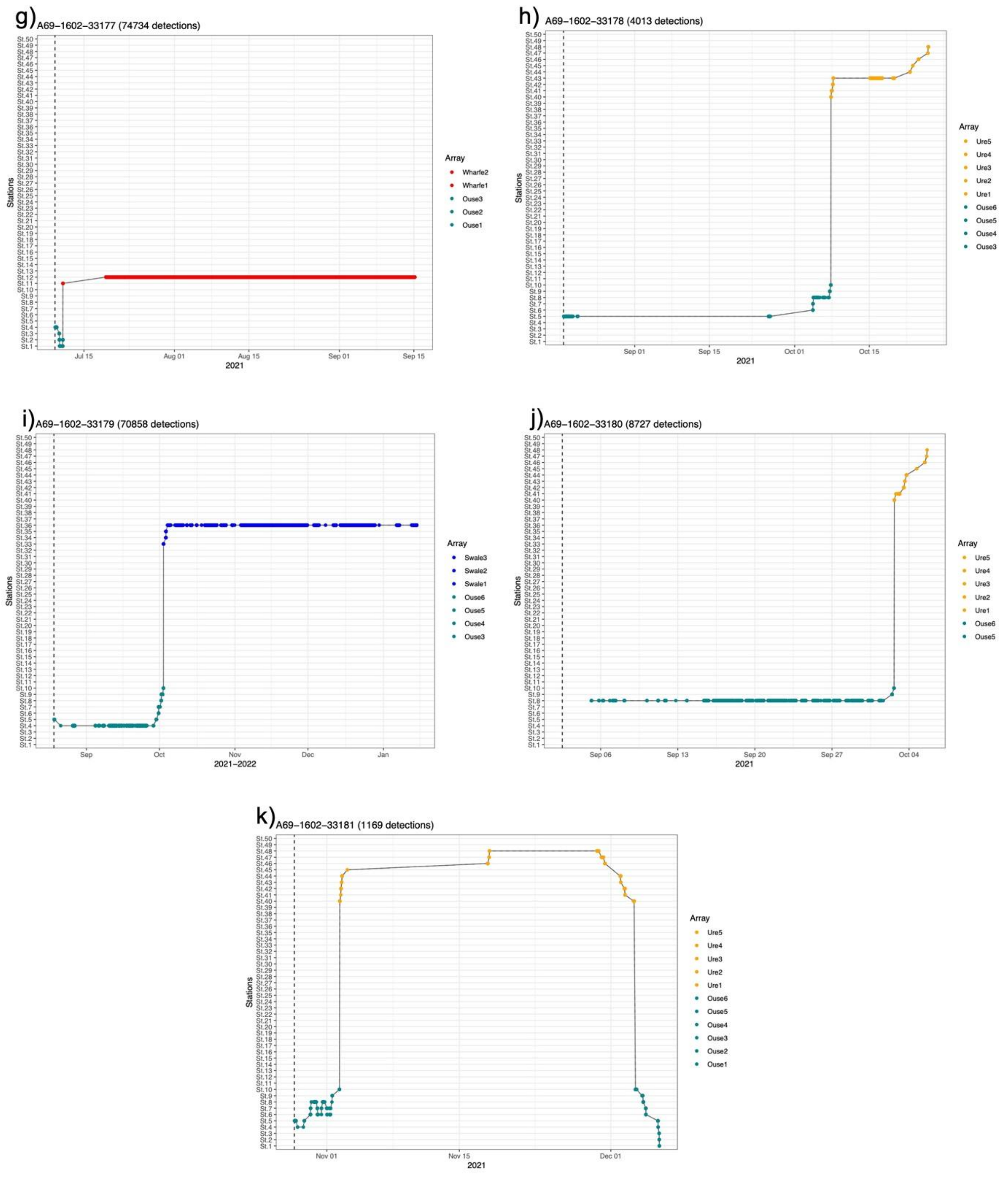

Supplementary Figure 5. Migration paths of the eleven returning Atlantic salmon tagged in the study. Three individuals (c, f, k) appeared to emigrate as kelts after spawning.

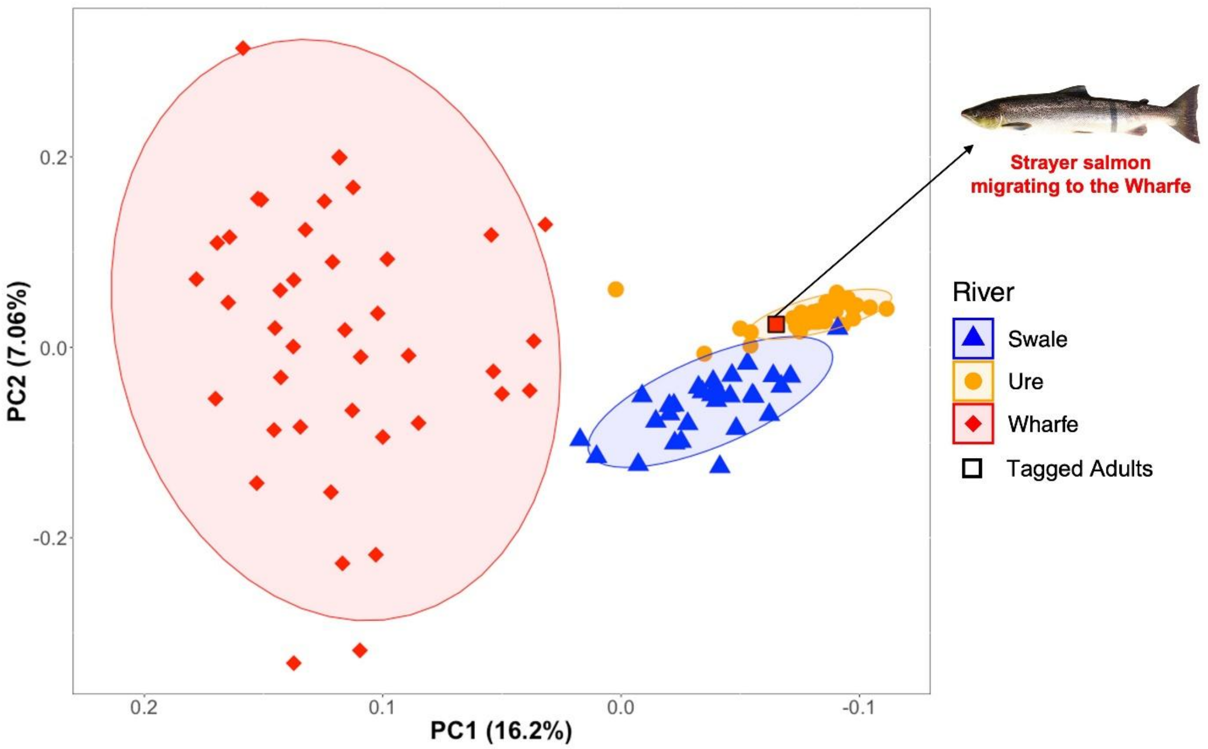

Supplementary Figure 6. Principal components analysis (PCA) based on 29,342 SNPs pruned for linkage disequilibrium. Symbols represent individual fish, and variance (%) explained by the first and second axes are shown. Colours correspond to rivers. The potentially straying returning adult fish is highlighted.

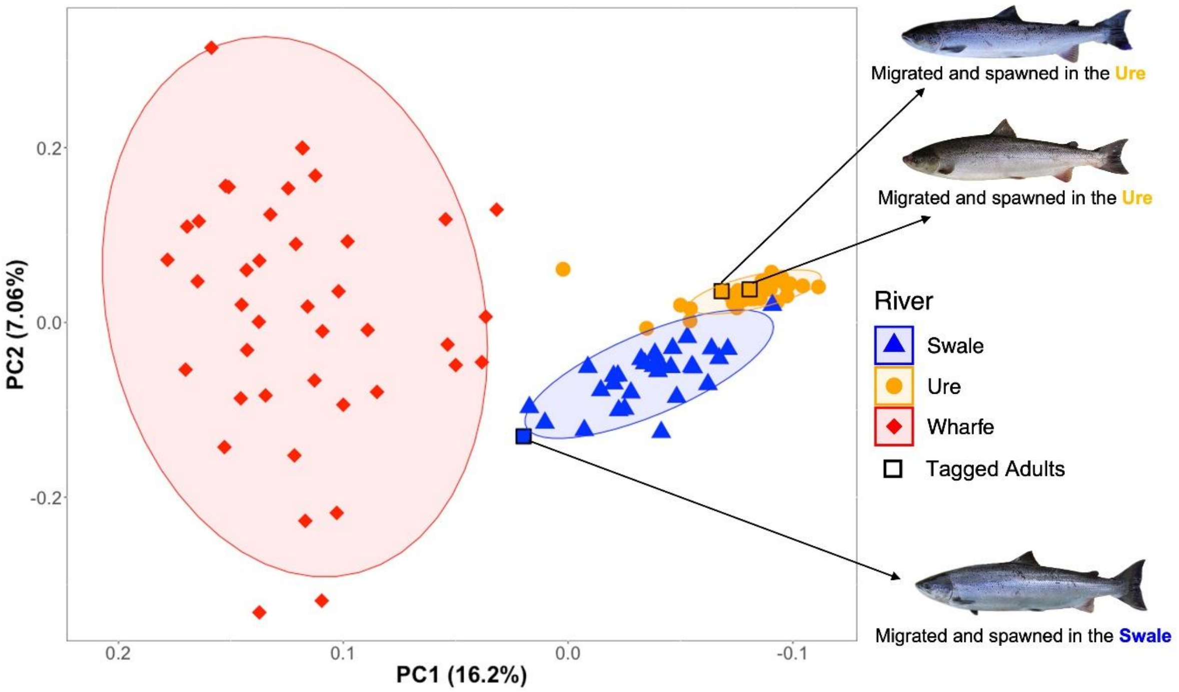

Supplementary Figure 7. Principal components analysis (PCA) based on 29,342 SNPs pruned for linkage disequilibrium. Symbols represent individual fish, and variance (%) explained by the first and second axes are shown. Colours correspond to rivers. Three immigrating Atlantic salmon showing exploratory behaviour before homing to their natal river to spawn are highlighted.

Supplementary Table 1. Numbers, lengths, weights and capture locations of adult salmon tagged in the Yorkshire Ouse.

Month	No.	Lengths (mm)	Weights (g)	Location(s)
March	0	–	–	–
April	0	–	–	–
May	0	–	–	–
June	4	750-820	4050-5000	Naburn + Linton-on-Ouse
July	2	800	4950-5200	Naburn
August	2	540-795	1225-4550	Naburn
September	2	577-730	1725-3150	Naburn + Linton-on-Ouse
October	1	960	7110	Naburn
November	0	–	–	–
**Total/Mean ± S.D.**	11	770 ± 99	4226 ± 1836	

Supplementary Table 2. Details of the acoustic receiver array used to monitor the migrations of tagged Atlantic salmon in the Yorkshire Ouse catchment.

Station	Latitude	Longitude	River	Array	Code
Cawood	53.833312	-1.1277404	Ouse	O1	St.1
Ouse DS Wharfe conf.	53.841922	-1.133808	Ouse	O2	St.2
Ouse US Wharfe conf.	53.843959	-1.1259537	Ouse	O2	St.3
DS Naburn Weir	53.892346	-1.0972354	Ouse	O3	St.4
US Naburn Weir	53.895925	-1.102241	Ouse	O3	St.5
Ouse DS Nidd conf.	54.014763	-1.2164173	Ouse	O4	St.6
Ouse US Nidd conf.	54.015572	-1.2191796	Ouse	O4	St.7
DS Linton weir	54.035723	-1.2374758	Ouse	O5	St.8
US Linton weir	54.033296	-1.2417341	Ouse	O5	St.9
DS Swale-Ure conf.	54.082114	-1.3385659	Ouse	O6	St.10
Wharfe US Ouse conf.	53.842058	-1.1364801	Wharfe	W1	St.11
DS Tadcaster Weir	53.885889	-1.261078	Wharfe	W2	St.12
US Tadcaster Weir	53.888548	-1.2637394	Wharfe	W2	St.13
DS Boston Spa Weir	53.904111	-1.3404819	Wharfe	W3	St.14
US Boston Spa Weir	53.908349	-1.3478131	Wharfe	W3	St.15
DS Flint Mill Weir	53.918474	-1.3554662	Wharfe	W4	St.16
US Flint Mill Weir	53.920728	-1.3617353	Wharfe	W4	St.17
DS Wetherby Weir	53.925324	-1.3830761	Wharfe	W5	St.18
US Wetherby Weir	53.926447	-1.3899581	Wharfe	W5	St.19
DS Harewood Weir	53.911378	-1.5218268	Wharfe	W6	St.20
US Harewood Weir	53.909694	-1.5346786	Wharfe	W6	St.21
DS Pool Weir	53.905389	-1.627619	Wharfe	W7	St.22
US Pool Weir	53.905652	-1.6523963	Wharfe	W7	St.23
DS Otley Weir	53.910866	-1.6889181	Wharfe	W8	St.24
US Otley Weir	53.907936	-1.6956374	Wharfe	W8	St.25
Nidd US Ouse conf.	54.012448	-1.2184751	Nidd	N1	St.26
DS Kirk Hammerton	53.983581	-1.2851833	Nidd	N2	St.27
US Kirk Hammerton	53.9867	-1.2866855	Nidd	N2	St.28
DS Hunsingore Weir	53.972614	-1.3448895	Nidd	N3	St.29
US Hunsingore Weir	53.971331	-1.3502911	Nidd	N3	St.30
DS Goldsborough Weir	53.999578	-1.4391255	Nidd	N4	St.31
US Goldsborough Weir	53.999234	-1.4405641	Nidd	N4	St.32
Swale US Ure conf.	54.089915	-1.3451535	Swale	S1	St.33
DS Crakehill Weir	54.151931	-1.3453851	Swale	S2	St.34
US Crakehill Weir	54.154562	-1.348161	Swale	S2	St.35
DS Topcliffe Weir	54.180308	-1.3921741	Swale	S3	St.36
US Topcliffe Weir	54.181324	-1.3958672	Swale	S3	St.37
DS Richmond Falls	54.404483	-1.729348	Swale	S4	St.38
US Richmond Falls	54.402432	-1.762727	Swale	S4	St.39
Ure US Swale conf.	54.090773	-1.3559033	Ure	U1	St.40
DS Boroughbridge Weir	54.097304	-1.3951922	Ure	U2	St.41
US Boroughbridge Weir	54.098281	-1.4017992	Ure	U2	St.42
DS Westwick Weir	54.094105	-1.4575604	Ure	U3	St.43
US Westwick Weir	54.098599	-1.4635726	Ure	U3	St.44
DS West Tanfield Weir	54.194432	-1.5610533	Ure	U4	St.45
US West Tanfield Weir	54.205011	-1.5813312	Ure	U4	St.46
DS Mickley Weir	54.18878	-1.6110871	Ure	U5	St.47
US Mickley Weir	54.186885	-1.6169127	Ure	U5	St.48
DS Aysgarth Falls	54.297034	-1.9643195	Ure	U6	St.49
US Aysgarth Falls	54.29348	-1.9899795	Ure	U6	St.50

Supplementary Table 3. Confidence intervals (97.5%) of pairwise genetic distance between tributaries of the Ouse as inferred by F_{ST}.

	**Swale**	**Ure**
**Ure**	0.014 - 0.015	
**Wharfe**	0.031 - 0.032	0.041 - 0.043

Supplementary Table 4. Pairwise Euclidean distances between the mean shapes of male Atlantic salmon from the Ouse tributaries. UCL = Upper Confidence Interval, Z = effect sizes.

**Comparison**	**Euclidean dist.**	**UCL (95%)**	***Z***	***P*-value**
Swale - Ure	0.013	0.009	3.410	0.0002
Swale - Wharfe	0.006	0.009	0.005	0.4956
Ure - Wharfe	0.013	0.008	4.160	0.0001
